# 2,4-Dichloro-6-[2-meth­oxy-4-(prop-2-en-1-yl)phen­oxy]-1,3,5-triazine

**DOI:** 10.1107/S1600536810042418

**Published:** 2010-10-30

**Authors:** Ya-Tuan Ma, Hong-Quan Li, Xin-Wei Shi, An-Ling Zhang, Jin-Ming Gao

**Affiliations:** a College of Science, Northwest A&F University, Yangling Shaanxi 712100, People’s Republic of China

## Abstract

The title compound, C_13_H_11_Cl_2_N_3_O_2_, was obtained by the reaction of eugenol and cyanuric chloride. The dihedral angle between the benzene and triazine rings is 87.56 (4)°. Two C atoms of the allyl group are disordered over two sites in a 0.72 (2):0.28 (2) ratio.

## Related literature

For background to the Williamson reaction in organic synthesis, see: Dermer (1934 ▶). For related structures, see: Ma *et al.*(2010*a* ▶,**b* ▶,c* ▶). For agricultural applications of the title compound, see: Manning *et al.* (1987 ▶).
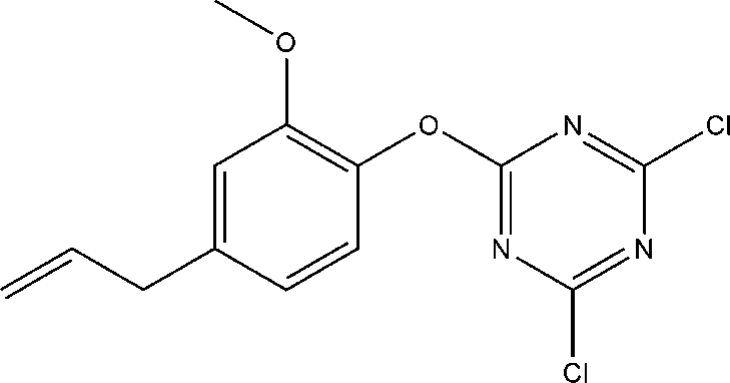


## Experimental

### 

#### Crystal data

C_13_H_11_Cl_2_N_3_O_2_
*M* *_r_* = 312.15
Monoclinic, 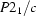

*a* = 11.4771 (12) Å
*b* = 8.6050 (9) Å
*c* = 14.7189 (13) Å
β = 103.077 (1)°
*V* = 1415.9 (2) Å^3^
*Z* = 4
Mo *K*α radiation
μ = 0.46 mm^−1^
*T* = 298 K
0.42 × 0.35 × 0.33 mm

#### Data collection

Siemens SMART CCD area-detector diffractometer
Absorption correction: multi-scan (*SADABS*; Sheldrick, 1996 ▶) *T*_min_ = 0.830, *T*_max_ = 0.862
6755 measured reflections
2499 independent reflections
1595 reflections with *I* > 2σ(*I*)
*R*_int_ = 0.027

#### Refinement

*R*[*F* ^2^ > 2σ(*F* ^2^)] = 0.038
*wR*(*F* ^2^) = 0.099
*S* = 1.02
2499 reflections
201 parameters
H-atom parameters constrained
Δρ_max_ = 0.23 e Å^−3^
Δρ_min_ = −0.23 e Å^−3^

### 

Data collection: *SMART* (Siemens, 1996 ▶); cell refinement: *SAINT* (Siemens, 1996 ▶); data reduction: *SAINT*; program(s) used to solve structure: *SHELXS97* (Sheldrick, 2008 ▶); program(s) used to refine structure: *SHELXL97* (Sheldrick, 2008 ▶); molecular graphics: *SHELXTL* (Sheldrick, 2008 ▶); software used to prepare material for publication: *SHELXTL*.

## Supplementary Material

Crystal structure: contains datablocks I, global. DOI: 10.1107/S1600536810042418/rn2069sup1.cif

Structure factors: contains datablocks I. DOI: 10.1107/S1600536810042418/rn2069Isup2.hkl

Additional supplementary materials:  crystallographic information; 3D view; checkCIF report

## Figures and Tables

**Fig. 1.**
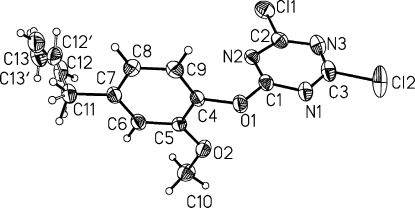
The molecular structure of (I), with atom labels and displacement ellipsoids drawn at the 30% probability level.The disordered atoms C12 and C13 of the allyl group are the major component.

## supplementary crystallographic information

### Comment

In this paper, we used the Williamson reaction (Dermer, 1934) to form the title compound, (I), which was synthesized by the reaction of eugenol, sodium hydroxide and cyanuric chloride at 278 K. We have previously reported three compounds of this type [Ma *et al.*(2010*a*, 2010*b* and 2010*c*)]. In (I)(Fig.1), the dihedral angle between the benzene ring C4—C9 and the triazine ring C2N3C3N1C1N2 is 87.56 (4)°. There are no significantly short intermolecular contacts in the crystal lattice.

### Experimental

492 mg eugenol (3 mmol) was dissolved in 1.2 g 10% sodium hydroxide (3 mmol) in a 100 mL round-bottom flask and the water was then removed *in vacuo*. Added 30 ml acetonitrile into the flask in an ice bath and after stirring 10 min cyanuric chloride (3 mmol) was added and kept stirred for 2 h at 278 K. The reaction mixture was filtered and solvent was evaporated under vacuum to dryness. The solid mass was dissolved in EtOAc, washed with saturated NaHCO_3_ and brine, dried over anhydrous Na_2_SO_4_, concentrated and purified with column chromatography to afford the crude product. White crystals suitable for X-ray analysis were obtained by slow evaporation of a solution of the title compound in n-hexane/ethyl acetate (1:1 V/V) at room temperature.

### Refinement

The atoms C12 and C13 were found to be disordered over two sites, and the ratio of the occupancy factors refined to 0.718 (7):0.282 (7) and 0.718 (7):0.282 (7) for atoms C12: C12' and C13: C13' respectively.The positions of all H atoms were determined geometrically and refined using a riding model with C—H = 0.93–0.97 Å and *U*_iso_(methyl H) = 1.5*U*_eq_(C) and 1.2*U*_eq_ for other H atoms.

### Crystal data

**Table:**

C_13_H_11_Cl_2_N_3_O_2_	*D*_x_ = 1.464 Mg m^−^^3^
*M**_r_* = 312.15	Melting point = 385–386 K
Monoclinic, *P*2_1_/*c*	Mo *K*α radiation, λ = 0.71073 Å
*a* = 11.4771 (12) Å	Cell parameters from 2205 reflections
*b* = 8.6050 (9) Å	θ = 2.8–25.7°
*c* = 14.7189 (13) Å	µ = 0.46 mm^−^^1^
β = 103.077 (1)°	*T* = 298 K
*V* = 1415.9 (2) Å^3^	Monoclinic, colourless
*Z* = 4	0.42 × 0.35 × 0.33 mm
*F*(000) = 640	

### Data collection

**Table:**

Siemens SMART CCD area-detector diffractometer	2499 independent reflections
Radiation source: fine-focus sealed tube	1595 reflections with *I* > 2σ(*I*)
graphite	*R*_int_ = 0.027
phi and ω scans	θ_max_ = 25.0°, θ_min_ = 1.8°
Absorption correction: multi-scan (*SADABS*; Sheldrick, 1996)	*h* = −13→12
*T*_min_ = 0.830, *T*_max_ = 0.862	*k* = −10→7
6755 measured reflections	*l* = −17→17

### Refinement

**Table:**

Refinement on *F*^2^	Primary atom site location: structure-invariant direct methods
Least-squares matrix: full	Secondary atom site location: difference Fourier map
*R*[*F*^2^ > 2σ(*F*^2^)] = 0.038	Hydrogen site location: inferred from neighbouring sites
*wR*(*F*^2^) = 0.099	H-atom parameters constrained
*S* = 1.02	*w* = 1/[σ^2^(*F*_o_^2^) + (0.0303*P*)^2^ + 0.7779*P*] where *P* = (*F*_o_^2^ + 2*F*_c_^2^)/3
2499 reflections	(Δ/σ)_max_ = 0.001
201 parameters	Δρ_max_ = 0.23 e Å^−^^3^
0 restraints	Δρ_min_ = −0.23 e Å^−^^3^

### Special details

**Table:**

Geometry. All e.s.d.'s (except the e.s.d. in the dihedral angle between two l.s. planes) are estimated using the full covariance matrix. The cell e.s.d.'s are taken into account individually in the estimation of e.s.d.'s in distances, angles and torsion angles; correlations between e.s.d.'s in cell parameters are only used when they are defined by crystal symmetry. An approximate (isotropic) treatment of cell e.s.d.'s is used for estimating e.s.d.'s involving l.s. planes.
Refinement. Refinement of *F*^2^ against ALL reflections. The weighted *R*-factor *wR* and goodness of fit *S* are based on *F*^2^, conventional *R*-factors *R* are based on *F*, with *F* set to zero for negative *F*^2^. The threshold expression of *F*^2^ > σ(*F*^2^) is used only for calculating *R*-factors(gt) *etc*. and is not relevant to the choice of reflections for refinement. *R*-factors based on *F*^2^ are statistically about twice as large as those based on *F*, and *R*- factors based on ALL data will be even larger.

### Fractional atomic coordinates and isotropic or equivalent isotropic displacement parameters (Å^2^)

**Table:**

	*x*	*y*	*z*	*U*_iso_*/*U*_eq_	Occ. (<1)
Cl1	0.23031 (7)	0.11286 (10)	0.56145 (6)	0.0754 (3)	
Cl2	0.08975 (7)	0.41854 (15)	0.81780 (6)	0.1061 (4)	
N1	0.29775 (19)	0.4567 (3)	0.78327 (14)	0.0541 (6)	
N2	0.36101 (18)	0.3198 (2)	0.66250 (14)	0.0459 (5)	
N3	0.16967 (19)	0.2701 (3)	0.69226 (16)	0.0621 (7)	
O1	0.47642 (15)	0.5029 (2)	0.75543 (11)	0.0524 (5)	
O2	0.47302 (16)	0.6829 (2)	0.60769 (12)	0.0583 (5)	
C1	0.3757 (2)	0.4227 (3)	0.73157 (17)	0.0450 (6)	
C2	0.2573 (2)	0.2488 (3)	0.64855 (18)	0.0525 (7)	
C3	0.1983 (2)	0.3770 (4)	0.7582 (2)	0.0613 (8)	
C4	0.5660 (2)	0.4821 (3)	0.70437 (17)	0.0439 (6)	
C5	0.5664 (2)	0.5829 (3)	0.63087 (17)	0.0433 (6)	
C6	0.6619 (2)	0.5747 (3)	0.58824 (17)	0.0486 (7)	
H6	0.6644	0.6411	0.5389	0.058*	
C7	0.7537 (2)	0.4693 (3)	0.61776 (18)	0.0499 (7)	
C8	0.7481 (2)	0.3691 (3)	0.6892 (2)	0.0567 (7)	
H8	0.8082	0.2958	0.7082	0.068*	
C9	0.6542 (2)	0.3762 (3)	0.73313 (19)	0.0543 (7)	
H9	0.6513	0.3090	0.7820	0.065*	
C10	0.4713 (3)	0.7868 (3)	0.5320 (2)	0.0654 (8)	
H10A	0.5382	0.8565	0.5478	0.098*	
H10B	0.3983	0.8456	0.5198	0.098*	
H10C	0.4763	0.7285	0.4774	0.098*	
C11	0.8576 (2)	0.4640 (4)	0.5703 (2)	0.0653 (8)	
H11A	0.8779	0.5693	0.5563	0.078*	0.72 (2)
H11B	0.9265	0.4199	0.6131	0.078*	0.72 (2)
H11C	0.8464	0.5438	0.5226	0.078*	0.28 (2)
H11D	0.9307	0.4883	0.6157	0.078*	0.28 (2)
C12	0.8333 (7)	0.3729 (13)	0.4838 (7)	0.066 (2)	0.72 (2)
H12	0.7661	0.3994	0.4382	0.079*	0.72 (2)
C13	0.8975 (13)	0.2594 (16)	0.4660 (11)	0.086 (3)	0.72 (2)
H13A	0.9654	0.2292	0.5099	0.103*	0.72 (2)
H13B	0.8759	0.2074	0.4093	0.103*	0.72 (2)
C12'	0.876 (2)	0.317 (2)	0.527 (2)	0.067 (6)	0.28 (2)
H12'	0.8899	0.2272	0.5634	0.080*	0.28 (2)
C13'	0.873 (3)	0.310 (5)	0.433 (2)	0.082 (8)	0.28 (2)
H13C	0.8587	0.3992	0.3970	0.099*	0.28 (2)
H13D	0.8844	0.2152	0.4060	0.099*	0.28 (2)

### Atomic displacement parameters (Å^2^)

**Table:**

	*U*^11^	*U*^22^	*U*^33^	*U*^12^	*U*^13^	*U*^23^
Cl1	0.0806 (5)	0.0678 (5)	0.0734 (5)	−0.0163 (4)	0.0081 (4)	−0.0086 (4)
Cl2	0.0585 (5)	0.1906 (12)	0.0795 (6)	−0.0079 (6)	0.0370 (4)	−0.0104 (7)
N1	0.0501 (13)	0.0681 (16)	0.0488 (13)	0.0005 (12)	0.0208 (11)	0.0046 (12)
N2	0.0491 (13)	0.0447 (13)	0.0449 (13)	−0.0015 (11)	0.0129 (10)	0.0040 (11)
N3	0.0480 (13)	0.0831 (19)	0.0547 (15)	−0.0086 (13)	0.0105 (11)	0.0101 (14)
O1	0.0533 (11)	0.0569 (12)	0.0531 (11)	−0.0097 (10)	0.0246 (9)	−0.0077 (9)
O2	0.0602 (11)	0.0621 (12)	0.0580 (12)	0.0142 (10)	0.0244 (9)	0.0081 (10)
C1	0.0452 (15)	0.0491 (17)	0.0422 (15)	0.0015 (13)	0.0127 (12)	0.0103 (13)
C2	0.0545 (16)	0.0548 (18)	0.0453 (15)	−0.0022 (15)	0.0052 (13)	0.0124 (13)
C3	0.0517 (17)	0.086 (2)	0.0493 (17)	0.0026 (17)	0.0182 (14)	0.0151 (17)
C4	0.0440 (14)	0.0464 (16)	0.0439 (15)	−0.0098 (13)	0.0155 (12)	−0.0053 (13)
C5	0.0459 (14)	0.0401 (15)	0.0452 (15)	−0.0030 (12)	0.0133 (11)	−0.0048 (13)
C6	0.0520 (15)	0.0490 (17)	0.0481 (15)	−0.0064 (14)	0.0177 (12)	0.0004 (13)
C7	0.0448 (15)	0.0508 (17)	0.0573 (17)	−0.0077 (14)	0.0181 (13)	−0.0059 (14)
C8	0.0484 (15)	0.0515 (18)	0.0705 (19)	0.0048 (14)	0.0142 (14)	0.0019 (15)
C9	0.0589 (17)	0.0510 (17)	0.0547 (17)	−0.0030 (15)	0.0165 (14)	0.0066 (14)
C10	0.0724 (19)	0.060 (2)	0.0636 (19)	0.0131 (17)	0.0160 (15)	0.0119 (17)
C11	0.0523 (17)	0.071 (2)	0.080 (2)	−0.0070 (16)	0.0292 (16)	−0.0036 (18)
C12	0.051 (3)	0.090 (5)	0.064 (4)	0.000 (3)	0.027 (3)	0.004 (4)
C13	0.074 (6)	0.086 (8)	0.108 (11)	−0.012 (5)	0.044 (6)	−0.021 (6)
C12'	0.068 (11)	0.068 (10)	0.078 (15)	0.013 (8)	0.043 (11)	0.010 (9)
C13'	0.074 (15)	0.10 (2)	0.081 (19)	−0.010 (14)	0.036 (13)	0.007 (12)

### Geometric parameters (Å, °)

**Table:**

Cl1—C2	1.711 (3)	C8—H8	0.9300
Cl2—C3	1.715 (3)	C9—H9	0.9300
N1—C3	1.312 (3)	C10—H10A	0.9600
N1—C1	1.332 (3)	C10—H10B	0.9600
N2—C2	1.312 (3)	C10—H10C	0.9600
N2—C1	1.330 (3)	C11—C12'	1.451 (18)
N3—C3	1.323 (4)	C11—C12	1.466 (7)
N3—C2	1.324 (3)	C11—H11A	0.9700
O1—C1	1.324 (3)	C11—H11B	0.9700
O1—C4	1.416 (3)	C11—H11C	0.9700
O2—C5	1.356 (3)	C11—H11D	0.9700
O2—C10	1.425 (3)	C12—C13	1.29 (2)
C4—C9	1.356 (4)	C12—H12	0.9300
C4—C5	1.387 (3)	C13—H13A	0.9300
C5—C6	1.382 (3)	C13—H13B	0.9300
C6—C7	1.384 (3)	C12'—C13'	1.38 (5)
C6—H6	0.9300	C12'—H12'	0.9300
C7—C8	1.372 (4)	C13'—H13C	0.9300
C7—C11	1.513 (3)	C13'—H13D	0.9300
C8—C9	1.378 (3)		
			
C3—N1—C1	112.3 (2)	H10B—C10—H10C	109.5
C2—N2—C1	112.5 (2)	C12'—C11—C12	34.3 (9)
C3—N3—C2	111.3 (2)	C12'—C11—C7	115.8 (6)
C1—O1—C4	119.25 (19)	C12—C11—C7	113.7 (3)
C5—O2—C10	117.7 (2)	C12'—C11—H11A	131.1
O1—C1—N2	120.1 (2)	C12—C11—H11A	108.8
O1—C1—N1	113.1 (2)	C7—C11—H11A	108.8
N2—C1—N1	126.7 (2)	C12'—C11—H11B	76.4
N2—C2—N3	128.4 (3)	C12—C11—H11B	108.8
N2—C2—Cl1	116.1 (2)	C7—C11—H11B	108.8
N3—C2—Cl1	115.5 (2)	H11A—C11—H11B	107.7
N1—C3—N3	128.7 (3)	C12'—C11—H11C	108.1
N1—C3—Cl2	115.7 (2)	C12—C11—H11C	77.4
N3—C3—Cl2	115.6 (2)	C7—C11—H11C	108.9
C9—C4—C5	122.0 (2)	H11A—C11—H11C	35.2
C9—C4—O1	120.0 (2)	H11B—C11—H11C	134.6
C5—C4—O1	117.7 (2)	C12'—C11—H11D	107.5
O2—C5—C6	125.5 (2)	C12—C11—H11D	132.7
O2—C5—C4	116.8 (2)	C7—C11—H11D	108.9
C6—C5—C4	117.7 (2)	H11A—C11—H11D	74.5
C5—C6—C7	121.1 (2)	H11B—C11—H11D	35.4
C5—C6—H6	119.4	H11C—C11—H11D	107.4
C7—C6—H6	119.4	C13—C12—C11	125.3 (14)
C8—C7—C6	119.2 (2)	C13—C12—H11C	140.6
C8—C7—C11	120.9 (3)	C11—C12—H11C	37.0
C6—C7—C11	119.9 (3)	C13—C12—H12	117.4
C7—C8—C9	120.6 (3)	C11—C12—H12	117.4
C7—C8—H8	119.7	H11C—C12—H12	91.6
C9—C8—H8	119.7	C12—C13—H13A	120.0
C4—C9—C8	119.4 (3)	C12—C13—H13B	120.0
C4—C9—H9	120.3	H13A—C13—H13B	120.0
C8—C9—H9	120.3	C13'—C12'—C11	120 (3)
O2—C10—H10A	109.5	C13'—C12'—H12'	119.9
O2—C10—H10B	109.5	C11—C12'—H12'	119.9
H10A—C10—H10B	109.5	C12'—C13'—H13C	120.0
O2—C10—H10C	109.5	C12'—C13'—H13D	120.0
H10A—C10—H10C	109.5	H13C—C13'—H13D	120.0
			
C4—O1—C1—N2	2.3 (3)	C9—C4—C5—C6	−1.4 (4)
C4—O1—C1—N1	−178.1 (2)	O1—C4—C5—C6	172.4 (2)
C2—N2—C1—O1	179.1 (2)	O2—C5—C6—C7	179.3 (2)
C2—N2—C1—N1	−0.5 (4)	C4—C5—C6—C7	0.2 (4)
C3—N1—C1—O1	179.5 (2)	C5—C6—C7—C8	1.5 (4)
C3—N1—C1—N2	−0.9 (4)	C5—C6—C7—C11	−179.5 (2)
C1—N2—C2—N3	1.6 (4)	C6—C7—C8—C9	−2.0 (4)
C1—N2—C2—Cl1	−179.54 (18)	C11—C7—C8—C9	179.0 (3)
C3—N3—C2—N2	−1.0 (4)	C5—C4—C9—C8	0.9 (4)
C3—N3—C2—Cl1	−180.0 (2)	O1—C4—C9—C8	−172.7 (2)
C1—N1—C3—N3	1.5 (4)	C7—C8—C9—C4	0.8 (4)
C1—N1—C3—Cl2	−177.49 (19)	C8—C7—C11—C12'	58.8 (16)
C2—N3—C3—N1	−0.7 (4)	C6—C7—C11—C12'	−120.3 (16)
C2—N3—C3—Cl2	178.3 (2)	C8—C7—C11—C12	96.6 (7)
C1—O1—C4—C9	−92.5 (3)	C6—C7—C11—C12	−82.5 (7)
C1—O1—C4—C5	93.6 (3)	C12'—C11—C12—C13	−23.9 (13)
C10—O2—C5—C6	1.2 (4)	C7—C11—C12—C13	−125.3 (10)
C10—O2—C5—C4	−179.6 (2)	C12—C11—C12'—C13'	26 (2)
C9—C4—C5—O2	179.4 (2)	C7—C11—C12'—C13'	121 (2)
O1—C4—C5—O2	−6.8 (3)		
